# Association of Interferon-Alpha and Ribavirin-Induced Thyroid Dysfunction with Severity of Disease and Response to Treatment in Pakistani Asian Patients of Chronic Hepatitis C

**DOI:** 10.1155/2012/864315

**Published:** 2012-09-02

**Authors:** Amina Nadeem, Muhammad Aslam

**Affiliations:** ^1^Department of Physiology, Army Medical College, National University of Sciences and Technology, Islamabad 44000, Pakistan; ^2^Department of Physiology, Shifa Tameer-e-Millat University, Islamabad 44000, Pakistan

## Abstract

*Objective*. To determine the association of thyroid dysfunction with the severity of the disease and response to treatment in patients of chronic hepatitis C. *Design*. Cohort study. *Patients*. One hundred and sixty seven noncirrhotic chronic hepatitis C patients were grouped into treatment group (*n* = 107) and control group (*n* = 60). *Measurements*. Baseline S. ALT and S. AST by IFCC and S. TSH, S. free T4, and S.T3 level were measured by chemiluminescence method. The severity of the disease was measured by Knodell histopathological index (HPI) on liver biopsy. Study group patients underwent 24-weeks IFN and ribavirin therapy and thyroid functions were determined at weeks 0, 12, and 24. Response to therapy was determined by PCR-HCV test. *Results*. 20 treated patients (18.69%) developed thyroid dysfunction with relative risk (RR) of 11.25 and attributable risk (AR) of 91%. Females were at higher risk. Hypothyroidism was common than hyperthyroidism. There was no significant association between thyroid dysfunction and severity of the disease (*P* = 0.81) and response to therapy (*P* = 0.79). *Conclusion*. Interferon-alpha and ribavirin therapy induces thyroid dysfunction in chronic hepatitis C patients. There is no association between severity of disease and response to therapy with interferon-induced thyroid dysfunction.

## 1. Background

According to studies carried out by the World Health Organization, approximately 170 million individuals (3% of the world's population) have been diagnosed to have chronic hepatitis C [1]. Chronic hepatitis C (CHC) accounts for 70–75% of all chronic hepatitis cases and 15–20% of all cases of cirrhosis of liver and hepatocellular carcinoma [2]. Interferon-alpha 2b (IFN-*α*) is recommended as the standard therapy for chronic hepatitis C in combination with ribavirin. IFN-*α* and ribavirin act by immunomodulation and antiviral mechanisms. They also have direct toxic effects on the thyroid [3]. Autoimmune disorders are associated with combination therapy and include diabetes mellitus, thyroid dysfunction, and skin diseases. Thyroid autoantibodies and thyroid disease are the most common autoimmune disorders associated with treatment [3–8]. 

The side effects of IFN therapy on thyroid gland are known for the last two decades. Manifestations of thyroid disease induced by combination therapy vary widely in frequency in different studies and have been reported to be in the range of 3.9–27.2% [3, 4, 8–19]. The types of thyroid disorder also vary and can manifest either as clinical autoimmune thyroiditis (i.e., Hashimoto's thyroiditis and Graves' disease) or as nonautoimmune thyroiditis (i.e., destructive thyroiditis and nonautoimmune hypothyroidism). Females and Asians are at higher risk, and hypothyroidism is more common than hyperthyroidism [10–19]. Association of thyroid dysfunction with degree of severity of disease judged on liver biopsy shows varying results in literature. In one study, it was found that aggravation of fibrosis has no association with the occurrence of thyroid autoimmunity and thyroid dysfunction [14] whereas another study showed that low fibrosis is significantly associated with an increased incidence of thyroid disorders [13]. Similarly, association of thyroid dysfunction with response to treatment does not have conclusive remarks in literature. Some studies showed better treatment response in presence of autoimmune hypothyroidism [20–22] while others concluded that nonautoimmune thyroid disorder is associated with better treatment response [23]. One study found no association between treatment response and occurrence of thyroid disease [24]. The present study was planned to assess the association of interferon- and ribavirin-induced thyroid dysfunction with the severity of the disease on liver biopsy and response to combination therapy. 

## 2. Objectives

The objective of the study is to determine the association of interferon-alpha, and ribavirin-induced thyroid dysfunction with severity of disease on liver biopsy and response to treatment in patients of chronic hepatitis C.

## 3. Operational Definitions

**Thyroid Dysfunction**

This encompasses both hyperthyroidism and hypothyroidism.

**Hyperthyroidism**

It is autoimmune hyperthyroidism. Autoantibodies are detectable in most cases.

**Hypothyroidism**

Nonautoimmune hypothyroidism or can be autoimmune Hashimoto's thyroiditis with detectable autoantibodies.

**Biphasic Thyroiditis**

Presence of hyperthyroidism at 12 weeks, followed by hypothyroidism at 24 weeks of therapy because of inflammatory process in thyroid gland, on radioisotope thyroid scan, increased uptake at 12 weeks and decreased uptake at 24 weeks of therapy.

## 4. Patients and Methods

This cohort study was conducted at Military Hospital, Rawalpindi, Pakistan from January 2006–February 2007. One hundred and twenty patients were initially screened for the study, out of which 13 patients were excluded due to the presence of cirrhotic changes in liver. A total of one hundred and sixty seven diagnosed noncirrhotic patients of chronic hepatitis C were included by nonprobability convenience sampling technique. The patients with cirrhotic changes on liver biopsy, prior history of treatment with IFN and/or ribavirin, history of preexisting thyroid disease, neoplastic, autoimmune, severe cardiac or pulmonary disease, currently using immunosuppressant and/ or steroids, and pregnancy were excluded from the study. The range of age was 18–48 years and both male, and female patients were included. The subjects were divided into treatment (*n* = 107) and control group (*n* = 60). The hepatitis C diagnosed cases in both treatment and control groups were included on the basis of persistently raised serum alanine transferase (ALT) by International Federation of Clinical Chemistry (IFCC) method on Selectra, positive Hepatitis C Virus (HCV) antibodies by 3rd generation Enzyme Linked Immuno Sorbent Assay (ELISA), qualitative positive HCV Ribonucleic Acid (RNA) by Polymerase Chain Reaction (PCR), and positive histopathological findings on liver biopsy (in treatment group only). Liver biopsies were performed under strict aseptic measures following the protocol. Necroinflammatory damage to liver parenchyma assessed on liver biopsy indicated severity of the disease. Liver biopsies were scored by Knodell Histopathological Index (HPI) based on inflammatory, necrotic, and fibrotic changes. Mild, moderate, and severe grades of the disease were given based on the scores. Both treatment and control group patients had normal baseline serum thyroid function depicted by serum thyroid stimulating hormone (S. TSH); reference range being 0.4–4.5 IU/L, serum-free thyroxine (S. Free T4); reference range being 8–24 pmol/L, and serum total triiodothyronine (S.T3) levels; reference range being 1.2–3 nmol/L by chemiluminescent on immulite 1000. Treatment group comprising of 107 patients were treated with Interferon alpha 2b (INF) three million units subcutaneously three times a week and ribavirin 800–1200 mg orally daily for 24 weeks. The control group comprising of 60 patients were chronic hepatitis C patients awaiting treatment subsequently. All biochemical variables described above were determined in treatment groups before the start of therapy, at 12 weeks during therapy, and at 24 weeks at the end of IFN therapy. 

Response to therapy was determined by qualitative HCV RNA by PCR test at the end of 24 weeks therapy in treatment group. In control group, S. ALT, S. TSH, S. Free T4, and S. total T3 were determined at weeks 0, 12, and 24. Mean and standard deviation of age, S. ALT, S. TSH, S. free T4, and S. total T3 were determined. Frequency of thyroid dysfunction was also determined. Relative risk (RR) ratio and attributable ratio (AR %) were calculated. Association between thyroid dysfunction and severity of disease was determined by applying chi-square test. Similarly, association between thyroid dysfunction and response to therapy was also determined by chi-square test. Statistical analyses were done on SPSS 15. Statistical significance was set at <0.05.

## 5. Results

One hundred and twenty patients were initially screened for the study, out of which 13 patients were excluded due to the presence of cirrhotic changes in liver. The treatment group patients (*n* = 107) met the inclusion criteria and were followed up till the end of 06 months treatment. The control group was also tested for all the biochemical parameters. The baseline serum ALT levels (Mean ± SD) and histology of liver biopsy are shown in Table 1. The ages ranged from 18–48 (35 ± 7.12) years and among them 80 were males and 27 were females. In treatment group, twenty patients, 10 females and 10 males (18.69%), developed thyroid dysfunction (either hyper or hypothyroidism) after 24 weeks of combination therapy with INF-alpha-2b and ribavirin therapy. In the control group (*n* = 60), 44 were males and 16 were females. Only one of the patients (1.66%) in control group developed subclinical hyperthyroidism (at the end of 24 weeks. In the treatment group, frequency of hypothyroidism was 10.2%; (60% of positive cases) while that of hyperthyroidism was 7.5% (40% of positive cases). Among hypothyroid cases, 2.8% of patients (15% of positive cases) exhibited transient hyperthyroidism at 12 weeks of therapy followed by hypothyroidism at the end of the treatment. These patients on thyroid scan showed increased radioisotope dye uptake initially at 12 weeks of therapy, when they manifested hyperthyroidism clinically and biochemically. On followup, thyroid scan showed decreased uptake of radioisotope dye at a time when patients were clinically and biochemically hypothyroid. Relative risk (RR) ratio was calculated to be 11.25 indicating patients of chronic hepatitis C undergoing treatment with INF and Ribavirin are eleven times more likely to develop thyroid dysfunction as compared to patients of chronic hepatitis C without treatment. The attributable risk (AR) % was 91% indicating that occurrence of thyroid dysfunction in patients of chronic hepatitis C is 91% attributable to INF and Ribavirin. None of the patients with thyroid dysfunction had to discontinue INF therapy. 

The histopathological changes in liver were subgrouped into mild, moderate, and severe disease on Knodell Histopathological Index scoring system (HPI). Out of 107 patients, a total of 51 (47.7%) had mild disease while 42 patients (39.3%) had moderate and 14 (13.1%) had severe disease on liver biopsy on the basis of Knodell HPI (Table 1). The relation between severity of the chronic hepatitis C disease and development of thyroid dysfunction was determined. Among 20 patients in treatment group who manifested features of thyroid dysfunction, 8 patients (40% of positive cases) had mild disease, 8 patients (40% of positive cases) had moderate disease, and 4 patients (20% of positive cases) had severe disease on Knodell HPI. Among 87 in treatment group whose thyroid functions remained normal clinically and biochemically, 43 patients (49.42% of negative cases), had mild disease, 34 patients (39.08% of negative cases) had moderate disease, and 10 patients (11.49% of negative cases) had severe disease on Knodell HPI. The difference between severity of the disease and development of thyroid dysfunction was not statistically significant (*P* value <0.81 on Chi-square test). 

In treatment group, the baseline ALT level was 93 ± 63 IU/L with a range of 13–383 IU/L and levels improved considerably with therapy. The ALT level during treatment at 12 weeks of therapy was 38.53 ± 30.86 IU/L with a range of 14–201 IU/L. At the end of treatment, the mean ALT level was 33.85 ± 24.02 IU/L and ranged between 11–170 IU/L. At 12 weeks of therapy, 88% of all the patients showed normal ALT levels and at the end of 24 weeks treatment, 97% of all the patients showed normalization of serum ALT levels. (*P* value at 12 weeks of therapy =0.001, and *P* value at 24 weeks of therapy =0.001 on Independent sample *t* test). Based on HCV RNA by PCR, among 107 patients of chronic hepatitis C, 92 patients (84%) showed remission at the end of 24 weeks treatment. The patients who did not respond to the therapy were males as well as females; in fact, 13.75% of males and 14.81% of females were nonresponders (Table 2). Response to therapy did not have any statistically significant relationship with sex of the patient (*P* = 0.89). Among 107 patients, 15 patients (16%) did not respond to therapy at the end of 24 weeks as predicted by positive HCV RNA by PCR. Among 20 patients who developed thyroid dysfunction, 17 (85%) responded to therapy and 03 (15%) did not respond to it. The association between thyroid dysfunction and response to therapy was also not significant statistically (*P* value =0.79 on Chi-square test) as shown in Table 2.

## 6. Discussion

The occurrence of thyroid dysfunction is the most common autoimmune response to interferon and ribavirin therapy. Though a number of studies have been carried out about the varying manifestation of thyroid disease and its pathogenesis, association of thyroid disease with severity of disease or response to treatment are reported with varying results. The therapeutic response to IFN and ribavirin therapy has been reported to be 70–80% in international [9–11] and national studies [12, 13, 18]. The response to IFN therapy is found to be associated with specific and nonspecific immunity against HCV [5, 9]. It was postulated that as HCV infection induces nonspecific immunity, it may lead to autoimmunity and thus increased risk of thyroid dysfunction along with better treatment response. Such associations have been reported in which presence of thyroid auto-antibodies and thyroid disease are correlated with better and sustained treatment response [20, 22, 25]. In one study, it was found that response rate to IFN therapy was better with absence of any auto antibodies, in nonautoimmune thyroid disease as compared to controls in the same cohort group [23]. Similar association has been reported in another study in which development of hypothyroidism in patients with thyroid autoantibodies undergoing treatment with IFN-alpha and Ribavirin was significantly associated with the long-term remission of CHC [26]. While other studies showed no such association either with IFN dosage, duration, or efficacy of therapy [4, 8, 15, 24, 27–29]. Morisco et al. [9] concluded that percentage of thyroid autoimmunity and thyroid dysfunction in long-term responders was not significantly different compared to that in nonresponders. In another study [18], development of thyroid disease was neither associated with dose of IFN nor with the viral kinetics nor virological response. The absence of cirrhosis was the only factor significantly related to successful response to therapy. In our study, no significant association was found in treatment response to IFN and ribavirin therapy with occurrence of thyroid disease (*P* value = 0.81). Probable explanations have been given in both the cases in which such association was present or absent. Activation of immunity leading to autoimmune manifestation along with better treatment response is hypothesized in case of positive associations. The probable reason for the negative association could be that the immune responses responsible for thyroid disease and those regulating therapeutic response are not the same. No definite answer is available yet although it is established that response to treatment depends on many factors including age and sex of the patient, viral load, viral genotypes, compliance to therapy, and degree of fibrosis in liver [14–16]. Adherence to therapeutic regimen, younger age, female sex, viral genotype 3, and lower body weight are factors associated with better treatment response of the patients. Interferon therapy induced thyroid disease cannot be considered as a predictive marker of therapeutic response. 

Literature review about association of thyroid disease with severity of the disease also shows varying results. In one study [30], patients with severe fibrosis on liver biopsy developed thyroid disease as compared to those with mild disease. In another study [9], it was found out that the occurrence of thyroid autoimmunity during interferon therapy was similar both in patients with or without worsening of liver disease (33.3% and 39.8%, resp.; *P* = not significant). The age was the only factor which was significantly associated with rapid progress of liver disease (odds ratio: 18.6; 95% confidence interval: 2.3–151.9, for those over 48 years versus younger patients). Other studies [4, 8, 31] reveal no association of thyroid dysfunctions with either dosage and duration of therapy or occurrence of thyroid autoimmunity and severity of disease. In our study, no association was found in severity of disease predicted on liver biopsy and occurrence of thyroid dysfunction. Severity of disease is governed by viral load, viral genotype, age, and sex of the patient and adherence to therapy. Thyroid dysfunction induced by interferon and ribavirin therapy is neither dependent on severity of the disease nor its prognostic marker.

The exact pathophysiology of INF-induced thyroid disease (IITD) is not known but presence of HCV itself augments the occurrence of IITD; the finding supported by the fact that higher dose of INF is given in HBV patients but results in lower incidence of IITD [32]. It is strongly suggested that HCV induces type 1 T helper cells (TH-1) responses leading to cellular immunity modification in genetically susceptible individuals culminating in IITD [33]. In addition to immune-modulation, inflammatory process and direct toxic effects of INF therapy in thyroid cell cultures and transgenic mice have been reported [34]. IFN treatment of cultured thyrocytes induces increased expression of thyroglobulin, sodium-iodide symporters, TSH receptors, and thyroid peroxidase [34]. IFN-induced infiltration of immune cells in thyroid gland leads to inflammatory destruction in addition to cellular immune response. Both processes lead to direct toxic effect on thyrocytes and immuno-modulation respectively, leading to IITD [34]. IFN induces JAK-STAT signaling pathway in thyroid cells and increases the expression of cytokine and adhesion molecule genes. In addition, it also increases MHC-I antigen on thyroid epithelial cells activating cytotoxic T cells [35]. The exact mechanism of immune modulation is not known but studies on thyroid follicle cell culture reveal that IFN-*α*, *β*, and *γ* induces TH-1 chemokines; CXCL-9 and CXCL-10 and TNF-*α* plays synergistic role [36]. The peroxisome proliferator-activated receptor-gamma, (PPAR-*γ*) agonists are found to partially inhibit this process in thyroid cell culture [36]. 

## 7. Conclusion

Although thyroid dysfunction is a common autoimmune adverse effect of interferon and ribavirin combination therapy, it can neither be considered as prognostic marker of the progressive fibrosis of liver nor as a predictive marker of therapeutic response. The immunological basis of the thyroid disease in chronic hepatitis C patients treated with interferon is still not clear which play role not only in the outcome of the thyroid dysfunction but also different manifestation of the disease. Further studies are needed to be done to understand the pathogenesis of interferon-induced thyroid disease, role of genetic, viral, and environmental factors in its etiology and outcome. Better knowledge of these aspects will lead to better understanding of the disease, improvement in patient care, earlier diagnosis of thyroid side effects, and more appropriate therapeutic approach to interferon-induced thyroid disease.

## Figures and Tables

**Table 1:** Thyroid dysfunction and severity of disease.

Thyroid function	Severity (no. of patients)			Total (*n*)
	Mild (*n*)	Moderate (*n*)	Severe (*n*)	
Euthyroid	43 (49.42%)	34 (39.08%)	10 (11.49%)	87
Hyperthyroid	03	03	02	08
Hypothyroid	03	04	02	09
Biphasic thyroiditis	02	01	0	03

Total	51 (47.66%)	42 (39.25%)	14 (13.08%)	107

*P* value <0.81 on chi-square test.

**Table 2:** Thyroid dysfunction and response to therapy.

Thyroid Function	PCR at end of treatment (no. of patients)		Total (*n* = 107)
	Negative	Positive	
Euthyroid	75 (70.09%)	12 (11.21%)	87
Hyperthyroid	07 (6.54%)	01 (0.93%)	08
Hypothyroid	07 (6.54%)	02 (1.86%)	09
Biphasic thyroiditis	03 (2.8%)	0 (0%)	03

Total	92 (83.98%)	15 (16.02%)	107

*P* value <0.79 on chi-square test.
